# A Randomised Controlled Single-Blind Trial of the Efficacy of Reiki at Benefitting Mood and Well-Being

**DOI:** 10.1155/2011/381862

**Published:** 2011-03-27

**Authors:** Deborah Bowden, Lorna Goddard, John Gruzelier

**Affiliations:** Psychology Department, Goldsmiths, University of London, ITC Builidng, New Cross, London SE14 6NW, UK

## Abstract

This is a constructive replication of a previous trial conducted by Bowden et al. (2010), where students who had received Reiki demonstrated greater health and mood benefits than those who received no Reiki. The current study examined impact on anxiety/depression. 40 university students—half with high depression and/or anxiety and half with low depression and/or anxiety—were randomly assigned to receive Reiki or to a non-Reiki control group. Participants experienced six 30-minute sessions over a period of two to eight weeks, where they were blind to whether noncontact Reiki was administered as their attention was absorbed in a guided relaxation. The efficacy of the intervention was assessed pre-post intervention and at five-week follow-up by self-report measures of mood, illness symptoms, and sleep. The participants with high anxiety and/or depression who received Reiki showed a progressive improvement in overall mood, which was significantly better at five-week follow-up, while no change was seen in the controls. While the Reiki group did not demonstrate the comparatively greater reduction in symptoms of illness seen in our earlier study, the findings of both studies suggest that Reiki may benefit mood.

## 1. Introduction

Reiki is a system involving the laying on of hands developed in Japan in the early 20th century [1] and is believed to have the capacity to heal the physical body and mind and bring emotional and spiritual balance. While the majority of scientific investigations have suffered from design limitations, however, there is some suggestive evidence that Reiki can influence mood [2–4] and induce physiological change in humans [5–10] and animals [11]. 

The present study employed a similar design to a previous study by the authors [4], where 35 first year undergraduates were randomly assigned to ten 20-minute sessions of Reiki or no-Reiki in conjunction with self-hypnosis/guided relaxation over a period of two and half to twelve weeks. While the Reiki group had a tendency towards a reduction of symptoms of illness following the intervention, a substantive increase in symptoms was seen in the no-Reiki group—leading to a highly significant distinction between them. There was also a trend for the Reiki group to have a greater improvement in overall mood than the no-Reiki group, accompanied by a near-significant comparative reduction in stress. However, the Reiki group had significantly higher baseline illness symptoms and mood scores than the no-Reiki group. The current study sought to replicate the comparatively greater mood and health benefits of the Reiki group in the previous study, while employing a design that ensured that the mean scores of the groups did not differ at baseline. In addition, the inclusion of participants with high depression and/or anxiety permitted the possibility that a greater degree of improvement could occur than was the case with the normally healthy participants of the first study.

## 2. Subjects and Methods

### 2.1. Participants

The study received approval from the Goldsmiths Ethics Committee prior to the recruitment of participants. 43 university students who were eligible for the study elected to participate, of ages ranging from 18–31 (except for one student aged 43) and of whom 32 were Psychology freshers. Only 40 students completed the study (37 females; 4 males) due to three drop-outs (all Reiki participants). The higher proportion of female participants was largely due to the high female-to-male ratio of Psychology undergraduates, and also perhaps because females were more inclined to take part. Of these 40 participants, 20 had high depression and/or anxiety with a Hospital Anxiety and Depression Scale (HADS) Anxiety or Depression subscale score of at least 10/20 or if the sum of these scores equalled 12/40 or more, and 20 had low depression and/or anxiety with HADS Anxiety and Depression scores both below 7/20 and a total score below 12/40. Following the distribution of information sheets to participants and obtaining their informed consent, participants were randomly assigned to the intervention groups. The students were awarded course credits or *£*10 and a Reiki session at the end of the study if participants were not in the Reiki group. Students taking medication for depression were not included.

### 2.2. Design and Procedure

The students with high depression and/or anxiety (High-Mood) and the participants with low depression and/or anxiety (Low-Mood) were randomly assigned to the Reiki or Control groups giving four subgroups: (1) High-Mood Reiki, (2) Low-Mood Reiki, (3) High-Mood Control, and (4) Low-Mood Control. 

A total of 43 participants were recruited over a period of four months and the three who withdrew from the study did so at an early stage when there were less than 10 participants per subgroup. The randomisation procedure consisted of the tossing of an unbiased coin to assign each new pair of High-Mood or Low-Mood participants to be recruited to the Reiki or Control groups, to ensure that there were equal numbers of participants in each group. If, for example, the first High-Mood participant to be recruited was randomly assigned to the Reiki group, then the next High-Mood participant was assigned to the Control group, and likewise with the Low-Mood participants until there were 10 participants in each of the four subgroups. When participants dropped out, new recruits continued to be randomly assigned to the four subgroups by the method described, until the target sample size was achieved.

G-Power was used to calculate the numbers of participants in the Reiki and Control groups needed to observe a significant difference between two independent samples of equal size. As with the study detailed in the previous chapter, it was predicted that the effect size would be of high magnitude, since similar or smaller sample sizes have been used in energy healing studies that have found significant effects [12]. Therefore, with an effect size of 1, an error probability of  .05, and an allocation ratio of 1, the necessary sample size was calculated to be 17 in each group. Thus, the 20 participants overall in each of the Reiki and Control groups were sufficient for an effect size of the predicted magnitude to be observed.

After completing questionnaires, as detailed in Psychological Measures, participants attended six half-hour treatment sessions. Due to the differing availability of participants, the period over which the six sessions were completed ranged from two to eight weeks, with one participant completing their sessions over 14 weeks. During each session both the Reiki and Control groups underwent a guided relaxation, where they listened to a 25-minute long audio file on headphones. The file consisted of 17-minutes of instructions designed to precipitate deep relaxation, followed by five minutes of peaceful nature sounds and music, concluded by instructions aimed to return participants to alertness. In addition to facilitating the blinding of participants to whether Reiki was being sent, the guided relaxation provided a control for the relaxation component of Reiki.

Questionnaires were again administered to participants approximately one week after the trial and again at five-week follow-up.

To aid relaxation, the treatment sessions were conducted in a dimly lit room where the participants reclined in comfortable chair with a foot rest. The conditions in the room and the interaction between the experimenter and participants were kept as constant as possible.

### 2.3. Reiki Method and Blinding

The Reiki in the present study was delivered by the experimenter who conducted the experimental sessions with participants. She had trained to Master-Teacher level in Usui Reiki in addition to having received attunements for Seichim, Violet Flame, and Ascension Reiki and had not trained in any other biofield modalities, and she had been practicing Reiki for four years. The experimenter used a combination of Reiki techniques, especially Ascension Reiki which was developed in 1998 by Wyllie and Mackenzie [13] where she used the Reiki symbols and techniques that she felt were most suitable for each participant.

A Reiki blinding technique was used that was successfully employed by the authors previously [4], where the experimenter sat behind each participant and sent noncontact Reiki to those in the Reiki group, whilst the participants' attention was absorbed in a task, here guided relaxation. All participants were informed at the outset that they may or may not receive noncontact Reiki. The experimenter sat roughly a metre behind each Reiki and Control participant during all experimental sessions, which were conducted with one participant at a time. She sent non-contact Reiki to those in the Reiki group, where her palms were positioned 3–30 inches above the participant's head or behind their back. In addition to the headphones worn by participants which blocked background sounds, the participants were blindfolded in order to prevent them noticing any shadows that may have been cast by the experimenter's hands.

## 3. Psychological Measures

### 3.1. Depression, Anxiety, and Stress Scale (DASS) [14]

The DASS21 is a 21-item mood questionnaire designed to measure negative emotional states of depression, anxiety, and stress, where respondents answer from 0 (not at all) to 3 (most of the time).

### 3.2. The Hospital Anxiety and Stress Scale (HADS) [15]

The HADS is a 14-item self-report measure designed to assess levels of Anxiety and Depression, where each item is scored on a scale of 0–21. Unlike the DASS, which was designed for use with both normal and clinical populations, the HADS was designed to assess the mood of hospital General Medical Outpatients, although it has been extensively used in Primary Care (Wilkinson and Barczak, 1998). 

### 3.3. Pittsburgh Quality of Sleep Index (PSQI) [16]

 The PQSI is a multi-item questionnaire that was used to assess several sleep components over the previous month including sleep disturbances, medication use, tiredness, and apathy. The postassessment version of the scale assessed sleep over the previous week in order for any effects of the intervention to manifest.

### 3.4. Illness Symptoms Questionnaire

 The ISQ was used to measure the presence of 20 illness symptoms such as fever, headache, and runny nose. Respondents stated the number of days in the past two weeks each symptom had been experienced. A score of 0 was assigned to a symptom present for zero days, a score of 1 for 1-2 days, a score of 2 for 3-4 days, a score of 3 for 5-6 days, and a score of 4 for 7–14 days.

### 3.5. Activation-Deactivation Adjective Check List (AD-ACL) [17]

 The AD-ACL measures items corresponding to Tension, Calmness, Energy, and Calmness. Participants rate how well a list of 26 adjectives (e.g., calm) describe how they are presently feeling on a scale of 1 (definitely do not feel) to 4 (definitely feel).

### 3.6. The Reiki Blinding and Expectation Questionnaire

A short questionnaire used previously by the authors [4] was completed before participants' fourth intervention session and again at Posttreatment to assess participants' beliefs regarding group membership and whether the intervention was benefitting their well-being. An answer of “no” received a score of 0, the answer “don't know” received a score of 1, and the answer “yes” corresponded to a score of 2.

### 3.7. Statistics

Mixed ANOVAs were used to compare the mean scores of the Reiki and Control participants for each of the measures completed preintervention (Baseline) and one-week (Posttreatment) and five weeks (Follow-up) following the intervention, as was proposed at the study outset. The within-subjects factor was Session^A^ (Baseline, Posttreatment, and Follow-up) and the between-subjects factors were Reiki-Group (Reiki or Control) and Mood-Group (High and Low). Then paired *t*-tests were conducted for each of the scales, comparing Baseline mean scores of the Reiki and Control groups separately with mean scores at Posttreatment and Follow-up. Two participants failed to return their Follow-up questionnaires and were not included.

For the AD-ACL which was completed before and after each of the six sessions, mixed ANOVAs were performed as with the other scales, but with the within-subjects factor of Session^B^ (Total Pre-Session and Total Post-Session), where Total corresponds to the sum of the AD-ACL scores of all six sessions.

## 4. Results

Before the intervention, only roughly half of the participants had heard of Reiki and only very small percentages had experienced Reiki before, and there were no statistical differences between the groups in these respects. 

### 4.1. Depression, Anxiety, and Stress Scale

Table 1 shows the means and standard deviations for the sum total of DASS items, Total DASS, and for the subscales Depression, Anxiety, and Stress. The outlying data of two participants were excluded from the DASS analysis—one Reiki participant had a Pre-Total DASS score that was 2.256 SDs above the sample mean and one Control participant had a Pre-Total DASS score that was 2.168 SDs above the sample mean.

For the sample as a whole, as can be seen from the total group means in Table 1, there was little change over the course of the study in the Total DASS. Accordingly, no significant main effect of Session was found with mixed ANOVA for the mean Total DASS score, or for Depression, Anxiety, or Stress, nor were there any Session × Reiki-Group effects (*F* ≤ 0.502, ns).

However, taking Reiki and Mood into account the mixed ANOVA showed significant three-way interactions between Session, Reiki-Group, and Mood-Group for Total DASS (*F* = 3.497, *P* = .036) and Anxiety (*F* = 3.149, *P* = .049) and Stress (*F* = 3.143, *P* = .05), while the interaction for Depression was nonsignificant (*F* = 1.651, *P* = .208). Before considering Posttreatment and Follow-up separately, importantly no statistical differences with independent samples *t*-tests were found between the Reiki and Control groups at Baseline, either overall or between the Reiki and Control participants of the High or Low-Mood Groups (*t* < 1.702, *P* > .108). It was thus reasonable to compare the changes in the mean DASS scores of the groups. 

**Figure 1:**
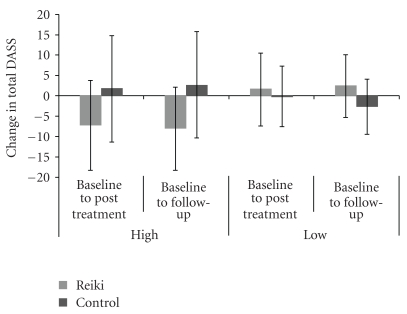
The Baseline to Posttreatment and Baseline to Follow-up changes in the mean Total DASS scores of the Reiki and Control participants of the High-Mood and Low-Mood groups, where a negative change corresponds to an improvement in mood.

Figure 1 shows the changes in the mean Total DASS scores that occurred over Baseline to Posttreatment and Baseline to Follow-up for the Reiki and Control participants of the High- and Low-Mood groups separately, where a negative change indicates an improvement in mood.

### 4.2. Posttreatment

Mixed ANOVA contrast analyses comparing Total DASS scores at Baseline and Posttreatment disclosed a tendency towards a Session × Reiki-Group × Mood-Group interaction (*F* = 3.166, *P* = .084). Separate mixed ANOVAs for the High- and Low-Mood groups showed for High-Mood participants a slight tendency towards a Session × Reiki-Group interaction (*F* = 3.285, *P* = .09) whereas Low-Mood participants did not differ (*F* = 1.03, ns). Paired *t*-tests with the High Mood groups indicated this was due to a greater improvement in Total DASS in the Reiki group, which was not seen in the control group (Reiki group mean change: 7.2/63, *t* = 2.217, *P* = .054; Control group mean change: 1.6/63; *t* = −0.033, ns). This can be seen in Figure 1.

### 4.3. Follow-Up

Contrast analyses comparing the Total DASS scores at Baseline and Follow-up disclosed a significant Session × Reiki-Group × Mood-Group interaction (*F* = 6.509, *P* = .016). As can be seen in Figure 1, there was a further reduction at Follow-up in the mean Total DASS score of the High-Mood Reiki participants, so that the mean was substantively lower than baseline (mean change: −8.1/63; Session × Group: *F* = 3.662, *P* = .075). This was verified by paired *t*-tests, which found a significant mean improvement in the Reiki group (*t* = 2.376, *P* = .045), which was not seen in the Controls.

**Figure 2:**
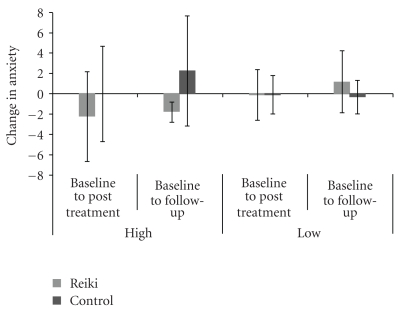
The Baseline to Posttreatment and Baseline to Follow-up changes in the mean Anxiety scores of the Reiki and Control participants of the High-Mood and Low-Mood groups, where a negative change indicates a reduction in anxiety.

Analysis of Anxiety in High-Mood participants also indicated a tendency towards a Session × Reiki-Group interaction (*F* = 3.423, *P* = .084), such that whereas with Reiki improvement was maintained at follow-up (Baseline: 7.2/21, Posttreatment: 5/21, Follow-up: 5.4/21), controls disclosed an increase in anxiety (Baseline: 5/21, Posttreatment: 5/21, Follow-up: 7.3/21). This can be observed in Figure 2. 

**Figure 3:**
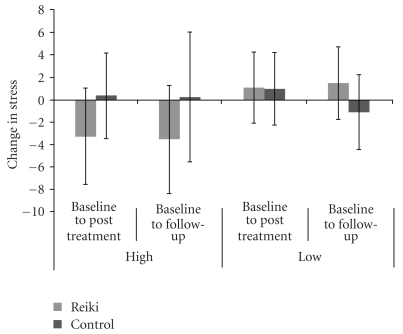
The Baseline to Posttreatment and Baseline to Follow-up changes in the mean Stress scores of the Reiki and Control participants of the High-Mood and Low-Mood groups, where a negative change corresponds to a decrease in Stress.

**Figure 4:**
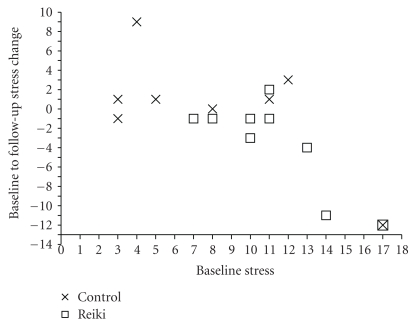
Scatter plot showing the Baseline to Follow-up Stress changes of the High-Mood Reiki and Control participants plotted against their Baseline scores, where the change scores of Reiki participants are denoted by boxes and those of Control participants by crosses, and a negative change corresponds to an improvement.

The greatest improvements in the High-Mood Reiki group at follow-up, however, were seen in the Stress subscale. As shown in Figure 3, there was a progressive improvement in the High-Mood Reiki participants, and at Follow-up their score was on average substantively lower than at Baseline (Baseline: 11.2/21, Follow-up: 7.7/21) (*t* = 2.223, *P* = .057). As can be seen from the mean scores shown in Figure 3 the High-Mood Control group was marginally worse at Follow-up compared to baseline, where only two participants had improved, while 5/8 had increased Stress scores. In contrast, 8/9 of the High-Mood Reiki group had reduced Stress. A Chi-squared test disclosed that the Reiki and Control groups differed significantly (*χ* ^2^ = 7.137, *P* = .008). The differential patterns of change of the two groups can be seen in Figure 4, which is a scatter plot showing the Baseline to Follow-up Stress changes of each of the High-Mood participants plotted against their Baseline scores, where a negative change corresponds to a decrease in Stress.

Regarding Depression, as can be seen from the group means in Table 1, so that it was markedly lower at Follow-up than at Baseline (Baseline: 7.4/21, Follow-up: 4.7/21) (*t* = 2.253, *P* = .054), whereas no change was seen in the High-Mood Control group.

### 4.4. The HADS, the PSQI, and the ISQ

The means and standard deviations for the sum total of items for the HADS (Total HADS), PSQI (Total PSQI), and ISQ (Total ISQ) are shown in Table 2. One Control participant with outlying data was excluded from the HADS analysis with a Posttreatment Anxiety score that was 3.25 standard deviations above the sample mean. 

As can be seen from the group means in Table 2, there was an improvement Posttreatment in Total HADS for the group as a whole **(**Session: *F* = 3.223, *P* = .046; contrast analyses: *F* = 4.757, *P* = .036). This was due to a reduction in the Anxiety subscale **(**Session: *F* = 4.618, *P* = .013; contrast analyses: *F* = 7.516, *P* = .01). However, these improvements were not maintained at Follow-up (Total HADS: *F* = 0.005, ns; Anxiety: *F* = 0.029, ns). No effect of Session was found for Depression (*F* = 0.715, ns).

Table 2 also shows an improvement in Global Sleep for the cohort as a whole (Session: *F* = 3.155, *P* = .049). However the trend for an improvement at Posttreatment, as indicated by contrast analyses (*F* = 3.51, *P* = .07), was not maintained at Follow-up. 

There was no change in the Total ISQ, however (Session: *F* = 0.028, ns).

Turning to the effects of Reiki, here there were no Session × Reiki-Group effects for Total HADS or for Anxiety or Depression, nor were there effects for the Total PSQI or Total ISQ (*F* ≤ 1.402, ns). There were also no significant interactions between Session, Reiki-Group, and Mood-Group (*F* ≤ 1.033, ns).

### 4.5. The Activation-Deactivation Adjective Check List

The means and standard deviations for the subscale of the AD-ACL are shown in Table 3.

Separate mixed ANOVAs were conducted for each of the AD-ACL subscales, finding for two of the subscales highly significant main effects of Session^B^ (Total PreIntervention-Session and Total PostIntervention-Session). There was a reduction in Tension (*F* = 42.017, *P* < .001) and an increase in Calmness (*F* = 34.781, *P* < .001) and Energy (*F* = 4.03, *P* = .052), although no effect was found for the Tiredness subscale (*F* = 0.604, ns). 

There were no Session × Reiki-Group or Session × Reiki-Group × Mood-Group effects for any of the AD-ACL subscales (*F* ≤ 0.776, ns).

### 4.6. Intersession Interval

In order to examine whether the time-length of the trial had an effect on its results, mixed ANOVAs were conducted with participants divided into groups of low (Low-Interval) and high (High-Interval) mean-intersession interval (MII). The distribution of MIIs of the Reiki and Control participants to the nearest day is shown in Table 4. As can be seen, 21/40 had an MII ranging from 3 to 5 days (mean: 4 days), which was taken to be the Low-Interval group (10 Reiki; 11 Control). Of the remaining 19/40—the High-Interval Group (11 Reiki; 9 Control)—18/19 had a MII ranging from 6 to 13 days (mean: 8.5 days), while that of the nineteenth member was 20 days.

Mixed ANOVAs were performed for each pre-post-assessment measure, where the between-subjects factors were Interval (High and Low) and Reiki-Group (Reiki and Control). No Session × Interval effects were found for any of the scales (*F* ≤ 1.7; ns). An independent samples *t*-test found that there was also very little difference between the MIIs of the Reiki and Control groups (*t* = −0.432, ns).

### 4.7. Reiki Blinding and Expectation Questionnaire

The Reiki and Control groups were very similar mid-intervention in their beliefs regarding their group-membership, as was confirmed by a Chi-Square test (*χ* ^2^(2,40) = 1.783, *P* = .41). At Posttreatment though, while equal numbers believed they had received Reiki (6/20 Reiki; 6/19 Control), more Controls believed they had not (6/20 Reiki; 11/19 Control), and more Reiki participants were unsure of their group (8/20 Reiki; 2/19 Control). This leads to a tendency for the groups to differ (*χ* ^2^(2,39) = 5.048, *P* = .08). However, since the majority of Reiki participants either believed that they were not in the Reiki group or were uncertain of their group, it seems that they could not detect the experimenter sending Reiki.

There was a substantive difference mid-intervention in the groups' beliefs about whether the trial was benefitting their well-being, where far more Reiki (14/20) than Control (3/30) participants were uncertain of this. Also, no Reiki participants believed that the intervention was benefiting them compared to 7/20 of the Controls, although conversely, more Control (10/10) than Reiki (6/10) participants were certain that it was not, leading to a significant distinction between the groups (*χ* ^2^(2,40) = 15.12, *P* = .001). There was no difference between the groups when participants completed the questionnaire at Posttreatment, however (*χ* ^2^(2,40) = 1.642, *P* = .44).

## 5. Discussion

The beneficial effects following Reiki found in this study for those participants with initially high levels of anxiety/depression, as evinced by the total Depression, Anxiety, and Stress Scale [14], are in keeping with the findings of our previous study [4]. There the Reiki group demonstrated comparatively greater overall mood and stress benefits than the controls who did not receive Reiki, accompanied by a buffering of the increase in symptoms of illness seen in the controls. 

Here the benefits were specific to those with high negative mood and were not found in the corresponding high negative mood control group. Posttreatment the total DASS score had improved with Reiki, and this was sustained over five weeks at follow-up. The main benefit was for the Stress subscale, which showed a mean four-scale point improvement at follow-up, where all but one of the Reiki participants had improved, whereas 5/8 control subjects showed an increase. These improvements were accompanied by reduced Anxiety of the order of two-scale point posttreatment and at follow-up, whereas the high negative mood controls showed an increase in Anxiety at follow-up (mean: two-scale points). For Depression, the mean score had dropped at Follow-up by three-scale points since baseline in the Reiki participants, while there was no change in the controls. These results are in accordance with the previous study of the authors, in which there were greater improvements in the total DASS and stress scores following Reiki [4]. Here though there were no baseline group differences favoring those who received Reiki as had occurred in the previous study, however, the preferential effects of Reiki on the high negative affect group found on the Depression Anxiety, and Stress Scale were not seen on the Hospital Anxiety and Depression scale [15], which focuses mainly on anhedonic depression [18]. Furthermore there was no benefit for Reiki on Illness Symptoms, unlike in our earlier study [4].

For the cohort as a whole an improvement in anxiety was found on the Hospital Anxiety and Depression Scale immediately following the intervention, which is in keeping with the guided relaxation that the participants received, although the improvement in Anxiety in the Depression, Anxiety and Stress Scale was not significant. In accordance with the reduction in HADS anxiety was the finding on the Pittsburg scale that the Global Sleep of the whole sample had improved posttreatment. The decrease in anxiety is also in keeping with the improvements in Calmness and Tension on the Activation-Deactivation checklist, although no change was seen on this scale in Tiredness. However, while the beneficial effects of Reiki on mood as evaluated by the DASS continued until five-week follow-up, neither the improvement in HADS anxiety, or Global Sleep for the cohort as a whole was maintained.

The Reiki-blinding method employed appeared to be successful. The majority of Reiki and Control participants both mid and post intervention either believed that they were not in the Reiki group (6/20 Reiki; 11/19 Control) or were not sure (8/20 Reiki; 2/19 Control), suggesting that participants were unable to detect the experimenter sending Reiki. While the study was limited by its lack of double-blinding, as the Reiki was administered by the experimenter who conducted the treatment sessions and in doing so interacted with participants, the experimenter was careful not to exert bias in her treatment of the Reiki and Control groups. The questionnaire replies suggested that this had been successful. 

Current and earlier studies are in keeping with the mood benefits observed in student populations following Johrei training whose healing practice is similar to Reiki, though does not require attunement [19, 20]. In one study the effects of stress were reduced in medical students who were randomised to groups who learned Johrei, self-hypnosis/visualisation, or relaxation training [19]. Whereas in the hypnosis and relaxation groups any decline in immune markers with exam stress was buffered for the groups as a whole, with Johrei all but one out of 12 participants showed an actual increases in CD3 − CD+ natural killer cell percentages with decreased percentages of CD3 + CD4. Benefits to mood in the form of reduced anxiety, depression, anger, and loss of vigour and confusion also followed Johrei training. The mood benefits observed in the current and previous studies also support the findings of a systematic review of those biofield therapies which are practiced proximally [12]. While there were insufficient numbers of studies included in the review to conduct an evidence-based synthesis of healthy participant populations or populations with mood disorders, moderate evidence was found that biofield therapies decrease anxiety in hospitalised populations. However, despite the growing body of evidence to support the efficacy of Reiki and other biofield therapies, many of the studies conducted to date have failed to effectively control for placebo. In addition, the vastly differing protocols employed paint an unclear picture of the factors required for efficacy, such as of the importance of touch, duration of interval between sessions, and the level of experience of the practitioner [21]. Clearly there is a need for rigorous, controlled research into the efficacy of biofield therapies that is built upon the current best evidence of clinical applications, as well as studies that investigate the effects of biofield therapies on specific biological and psychological processes. Considering our two controlled studies as a whole the benefits for symptoms of illness and the replicable benefits for mood should encourage further investigation.

## Figures and Tables

**Table 1:** Means (SD)* of total DASS, depression, anxiety, and stress**.

		Reiki group			Control group			Sample total
		Total	High-Mood	Low-Mood	Total	High-Mood	Low-Mood	
Total DASS	Baseline	17	25.9	8.9	14.4	19.4	10.5	15.7
		10.7*	(6.4)	(6.5)	(9.6)	(9.8)	(7.8)	(10.1)
	Posttreatment	14.4	18.7	10.5	15.1	21.1	10.3	14.7
		(10.2)	(10.8)	(8.2)	(10)	(8.3)	(8.4)	(10)
	Follow-up	14.4	17.8	11.4	14.2	22.1	7.8	14.3
		(9.1)	(8.1)	(9.3)	(12.1)	(14.1)	(4.6)	(10.5)

Depression	Baseline	5.1	7.4	2.9	5.7	6.5	5	5.4
		(4.2)	(5.7)	(2.3)	(4.8)	(6.2)	(3.6)	(4.4)
	Posttreatment	4.3	5.3	3.3	5.7	7.9	4.3	5.1
		(4.1)	(4.7)	(3.5)	(4.9)	(4)	(5.3)	(4.6)
	Follow-up	3.6	4.7	2.7	5	6.8	3.6	4.3
		(3.5)	(4.1)	(2.9)	(4.4)	(5.3)	(3.1)	(4)

Anxiety	Baseline	4.6	7.2	2.3	3.2	5	1.7	3.9
		(3.7)	(3.2)	(2.3)	(3.1)	(3.5)	(1.8)	(3.5)
	Posttreatment	3.5	5	2.2	3.1	5	1.6	3.3
		(3.5)	(3.5)	(3)	(3.3)	(3.7)	(2.3)	(3.4)
	Follow-up	4.2	5.4	3.5	4	7.3	1.4	4.2
		(3.2)	(2.8)	(3.4)	(4.8)	(5.7)	(1.3)	(4.1)

Stress	Baseline	7.3	11.2	3.7	6.5	7.9	3.8	6.5
		(5)	(3.1)	(3.5)	(4.8)	(5.1)	(3.3)	(4.8)
	Posttreatment	6.3	8	4.8	6.3	8.3	4.8	6.3
		(4)	(4.2)	(3.3)	(3.8)	(4.1)	(2.7)	(3.8)
	Follow-up	6.4	7.7	5.2	5.8	8.1	2.7	5.8
		(3.6)	(3)	(3.8)	(4)	(4.7)	(2.2)	(4)

*Standard deviations are shown in parentheses. **A lower score indicates better mood.

**Table 2:** Means and standard deviations* of total HADS, total PSQI, and total ISQ**.

		Reiki group	Control group	Sample total
Total HADS	Baseline	9.56	11.79	10.7
		(6.14)*	(6.72)	(6.54)
	Posttreatment	8.28	10.2	9.29
		(4.48)	(6.14)	(5.44)
	Follow-up	9.72	12.11	10.95
		(4.74)	(6.19)	(5.56)

Total PSQI	Baseline	10.7	9.63	10.15
		(2.71)	(3.24)	(3)
	Posttreatment	9.32	9.56	9.44
		(3.03)	(3.16)	(3.06)
	Follow-up	10.5	10.16	10.33
		(2.97)	(3.01)	(2.95)

Total ISQ	Baseline	11.47	10.67	11.08
		(6.68)	(9.59)	(8.12)
	Posttreatment	10.95	10.53	10.74
		(9.96)	(10.16)	(9.92)
	Follow-up	12.16	9.94	11.08
		(10.87)	(7.49)	(9.32)

*Standard deviations are shown in parentheses. **A lower score indicates better mood.

**Table 3:** Mean and standard deviations* Pre- and Post-Session scores of the AD-ACL subscales**.

	Tension		Calmness		Energy		Tiredness	
	Pre	Post	Pre	Post	Pre	Post	Pre	Post
Reiki group	55.50	44.85	75.45	90.10	75.15	69.15	69.80	67.60
	(18.00)*	(15.93)	(11.91)	(9.60)	(17.41)	(14.90)	(16.41)	(14.41)

Control group	54.75	40.75	72.00	85.80	67.55	94.59	73.25	70.30
	(13.47)	(8.26)	(13.83)	(11.64)	(16.69)	(25.84)	(15.32)	(18.99)

Sample total	55.13	42.80	73.72	87.95	71.35	97.57	71.52	68.95
	(15.70)	(12.69)	(12.86)	(10.74)	(17.27)	(26.34)	(15.77)	(16.73)

*Standard deviations are shown in parentheses. **A higher score corresponds to the greater presence of a symptom.

**Table 4:** Distribution of the mean intersession-intervals of participants in days.

	Low-interval			High-interval								Sample total
	3	4	5	6	7	9	10	11	12	13	20	
Reiki group	5	3	2	3	2	0	1	2	0	0	0	20
Control group	2	6	3	1	2	1	3	0	2	1	1	20
Sample total	7	9	5	4	4	1	4	2	2	1	1	40
